# Motor imagery as a cognitive mechanism in interventions for children with developmental coordination disorder: a narrative review

**DOI:** 10.3389/fped.2026.1745135

**Published:** 2026-05-18

**Authors:** Yiheng Chi, Li Ke, Tanghai Cheng, Xinrui Ma

**Affiliations:** State Key Laboratory of Cognitive Neuroscience and Learning, Beijing Normal University, Beijing, China

**Keywords:** child psychiatry, cognitive intervention, cognitive mechanism, developmental coordination disorder, motor imagery

## Abstract

**Introduction:**

Developmental Coordination Disorder (DCD) is a prevalent neurodevelopmental disorder characterized by significant motor impairments. Traditionally viewed as a deficit in motor execution, it is now increasingly understood to involve disruptions in cognitive processes underpinning motor control, including internal modeling, motor planning, and predictive control. This cognitive reconceptualization suggests the need for a shift in intervention approaches.

**Methods:**

This narrative review synthesizes theoretical, neurophysiological, and empirical literature to critically evaluate the role of Motor Imagery (MI)—the mental simulation of action without overt movement—as a cognitive mechanism for intervention in children with DCD. We examine the core cognitive and representational deficits in DCD, outline the neural foundations and theoretical frameworks of MI, and provide a narrative synthesis finding from key intervention studies.

**Results:**

Evidence suggests that children with DCD often exhibit impairments in motor imagery ability, reflecting possible disruptions in internal modeling processes. Nonetheless, structured MI-based interventions, particularly when combined with action observation (AOMI), have shown promising, though preliminary, effects in improving motor performance and activities of daily living. MI has been shown to engage neural networks overlapping with those involved in motor execution, and may promote neuroplasticity and support the perception-action cycle by facilitating predictive control and sensorimotor integration.

**Conclusion:**

MI may represent not only a therapeutic technique but also a useful window for understanding the cognitive mechanisms underlying DCD. By potentially targeting impaired internal models, MI-based approaches may contribute to functional improvements, although direct causal evidence remains limited. Future research should focus on standardize methodologies, conduct larger-scale trails, and carefully examine emerging technologies to develop personalized and ecologically valid intervention protocols. We propose a forward-looking perspective in which MI may serve as a component of mechanism-driven, technology-augmented, and ecologically valid interventions, potentially contributing to a shift from compensatory training toward more active cognitive-oriented approaches in DCD rehabilitation.

## 1 Introduction

Developmental Coordination Disorder (DCD) is a common yet often underestimated neurodevelopmental disorder primarily affecting children, with prevalence estimates generally ranging from 5% to 20%. It manifests as pronounced difficulties in coordinating voluntary movements, often leading to poor handwriting, clumsiness, and reduced participation in physical and social activities (1–3). Beyond its motor manifestations, DCD is associated with substantial impacts on self-esteem, academic performance, and psychosocial well-being (4, 5).

For decades, DCD was primarily conceptualized as a disorder of motor execution, in which deficits in muscle control or timing were believed to be central (6, 7). However, accumulating evidence from behavioral, neurophysiological, and neuroimaging studies has challenged this view. Children with DCD exhibit impairments in internal modelling, motor planning, and executive control, suggesting that motor deficits may be related to disruptions in higher-order cognitive representations that guide movement (8–10). In other words, their difficulties in performing coordinated actions are not purely mechanical but may involve impairments in processes, such as prediction, planning, and feedback monitoring.

This cognitive perspective has important implications for intervention. Traditional motor training, which focuses on repetitive physical practice, often results in limited or poorly generalized gains (11). In contrast, interventions that explicitly engage cognitive mechanisms, such as strategy-based training and mental simulation, have been associated with more lasting improvements (12–15). These findings suggest that an important determinant of a motor intervention's effectiveness may lie in its capacity to engage and reorganize the cognitive processes supporting motor control.

Within this framework, Motor Imagery (MI) has garnered growing attention as a theoretically grounded approach that may support motor learning in DCD (15–18). MI involves the internal simulation of an action without physical execution, enabling individuals to rehearse movement sequences, refine sensorimotor predictions, and potentially strengthen neural representations of coordinated actions (19, 20).

MI engages neural network that overlap with those involved in movement execution, including the premotor cortex, supplementary motor area, parietal lobules, and cerebellum, suggest that it may serve as a powerful cognitive proxy for motor practice (21). In children with DCD, where predictive control and sensory integration are often impaired, MI may offer a means of facilitating cognitive aspects of motor preparation prior to execution.

Despite these promising insights, MI-based interventions for DCD remain underexplored and heterogeneous in design. Key questions persist: What mechanisms mediate MI's effects on motor performance? How do age, imagery vividness, or task type modulate training outcomes? To what extent can MI training transfer to real-world functional gains? And how can emerging technologies, such as virtual reality (VR) and wearable sensing devices, be leveraged to enhance engagement and ecological validity?

This narrative review aims to address these questions by integrating theoretical, empirical, and applied perspectives on MI in DCD. We first outline the cognitive and representational deficits underlying DCD, then examine the neural and psychological foundations of MI. Next, we synthesize existing evidence on MI-based interventions, identify key moderators and limitations, and propose future directions for developing mechanism-driven, technology-augmented, and personalized MI interventions. Through this synthesis, we aim to position MI not merely as a therapeutic technique but also a conceptual lens for understanding the cognitive foundations of motor control in DCD.

## 2 Cognitive and representational deficits in DCD

Extensive research suggests that the motor difficulties experienced by children with developmental coordination disorder are not only related to inefficient movement execution but may also be associated with impairments in cognitive and representational systems. Early research suggested that DCD may be associated with diffuse brain dysfunction rather than abnormalities in specific brain regions. They grouped DCD alongside dyslexia and ADHD under the label of “atypical brain development” (ABD) (22). Another prominent hypothesis based on DCD motor control deficits concerns the internal models of movement. According to the Internal Modeling Deficit Hypothesis (23), individuals require an internal model to predict the sensory feedback of movements during motor planning. Following execution, they adjust the action plan based on error information (24). Children with DCD exhibit impairments in the action prediction and feedback processing, which may result in unstable movement planning, impaired timing, and reduced ability to utilize sensory feedback for correction (5).

Neuroimaging evidence provides support for this perspective. Functional magnetic resonance imaging (fMRI) and electroencephalography (EEG) studies have shown that children with DCD exhibit lower activation levels in regions such as the parietal lobe, premotor cortex, and cerebellum during movement execution or imagery compared to typically developing peers (25–28). These regions are considered important for sensorimotor integration and internal prediction processes (29–31). Their altered activity may reflect difficulties in integrating sensory input into motor output in children with DCD. Furthermore, weakened cerebellar-cortical loop connectivity is associated with impairments in motor timing control and error correction (32).

At the cognitive processing level, children with DCD also exhibit widespread impairments in executive function and working memory, particularly in motor planning, inhibitory control, and strategy switching (31, 33–35). Deficits in these higher-order cognitive functions may further contribute to coordination difficulties in complex tasks. For instance, in tasks requiring multi-step action planning or environmental adaptation, children with DCD often show difficulties in forming coherent action representations or dynamically adjusting strategies based on feedback (36–39).

Additionally, children with DCD tend to perform worse than controls in motor imagery and action observation tasks. They exhibited slower response times and lower accuracy in tests such as mental rotation, gesture judgment, and imagery temporal consistency, suggesting limited accessibility of internal representations (8, 40–42). In other words, they not only experience difficulties in performing actions but also show inefficiencies in perception-to-movement mapping.

**Figure 1:**
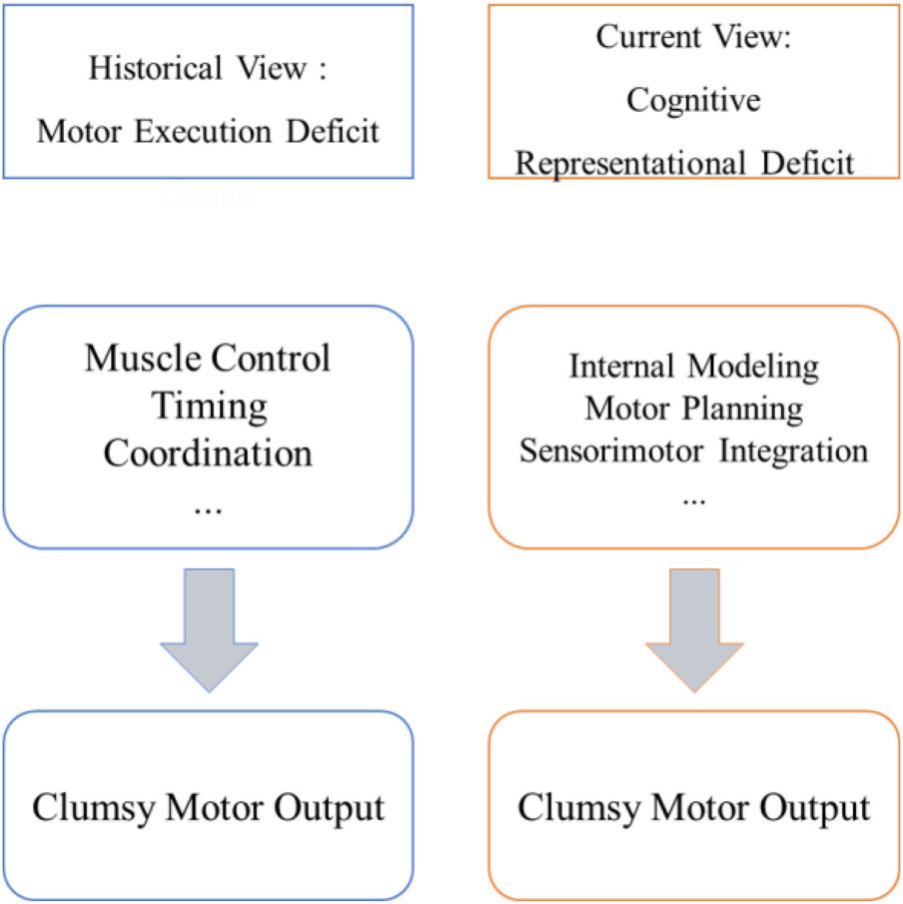
A paradigm shift in the conceptualization of developmental coordination disorder (DCD). The historical view framed DCD as a deficit in motor execution, such as problems with muscle control and timing. The contemporary cognitive perspective identifies the core impairment as a disruption in higher-order cognitive and representational systems, including internal modeling, motor planning, and sensorimotor integration, which guide and predict movement. This figure is a conceptual summary adapted from current literature.

In summary, the core of DCD lies not in isolated motor execution deficits but in comprehensive impairments of the cognitive and motor representation system (see Figure 1). This impairment encompasses both the accumulation of prediction errors in internal models and inefficiencies in executive functions and representational updating mechanisms. Physical training alone may be insufficient to fully address their underlying cognitive deficits. Instead, it may be beneficial to engage motor representations at the cognitive level to reconstruct the perception and executive pathway and restore predictive control capabilities. This cognitive orientation provides a robust theoretical foundation for subsequent intervention studies.

## 3 Cognitive components in motor interventions: theoretical foundations

As understanding of DCD mechanisms deepens, researchers increasingly recognize that cognitive processing plays a central role in motor learning and rehabilitation interventions. Traditional process-oriented training primarily emphasizes movement decomposition and sensory stimulation, such as Sensory Integration Therapy or repetitive motor practice. However, these interventions often yield limited effects in functional transfer and maintenance (14, 43). In contrast, cognitive-oriented interventions, which emphasize individual active thinking, planning and self-monitoring, have been demonstrated in recent years to produce more enduring improvements (3).

The core tenet of the cognitive orientation is that motor skill acquisition depends not only on physical practice but also on an individual's ability to form effective internal representations and motor plans (44). From this perspective, motor control is viewed as a closed-loop system comprising “perception-cognition-action.” Before executing an action, individuals must cognitively process information to predict outcomes, select appropriate movement strategies, and then adjust based on feedback after execution (45).

The most widely applied cognitive orientation model is the CO-OP (Cognitive Orientation to daily Occupational Performance) approach (46). This method emphasizes guiding children through the “Goal-Plan-Do-Check” cycle to actively consider action strategies, monitor performance outcomes and develop transferable cognitive templates, thereby promoting the active use of memory, attention, and mental planning (47). Research indicates that CO-OP not only improves performance on specific tasks but also promotes cross-task transfer and enhances daily functioning (48–50). This suggests that the effectiveness of the intervention may depend in part on the cognitive processes engaged during training. The importance of cognitive processing in motor interventions is also supported by neuroscience. Functional imaging studies reveal that during effective motor learning, individuals activate not only the motor cortex but also significantly engage prefrontal, parietal, and cerebellar networks-areas closely associated with executive function, planning, and working memory (51–53). Thus, motor learning can be understood as involving substantial cognitive processes, with movement improvement serving as an external manifestation of reorganized cognitive representations.

In DCD rehabilitation practice, cognitive-guided strategies have been demonstrated to enhance the sustainability and generalization of interventions (48, 54). Unlike passive imitation or mechanical repetition, cognitive training prompts children to form mental representations of movements through thinking, imagination, and self-guidance, enabling predictive control during execution (46). This approach provides the theoretical foundation for higher-level intervention forms such as motor imagery and action observation (18).

Overall, the effectiveness of motor interventions may depend on the extent to cognitive processes are engaged rather than merely repeating movements. Cognitive mechanisms may contribute to the reconstruction of impaired internal models of children with DCD by optimizing action planning, monitoring, and feedback updates. This may support more flexible and autonomous motor control without relying on external cues. This theoretical foundation provides a robust logical basis for subsequent MI-based intervention research.

## 4 Motor imagery: conceptual and mechanistic framework

Motor Imagery (MI) refers to the mental simulation of an action without overt execution, allowing individuals to represent key temporal and spatial features of movement in the absence of actual motor output (19, 20).

### 4.1 Types and characteristics of motor imagery

**Figure 2:**
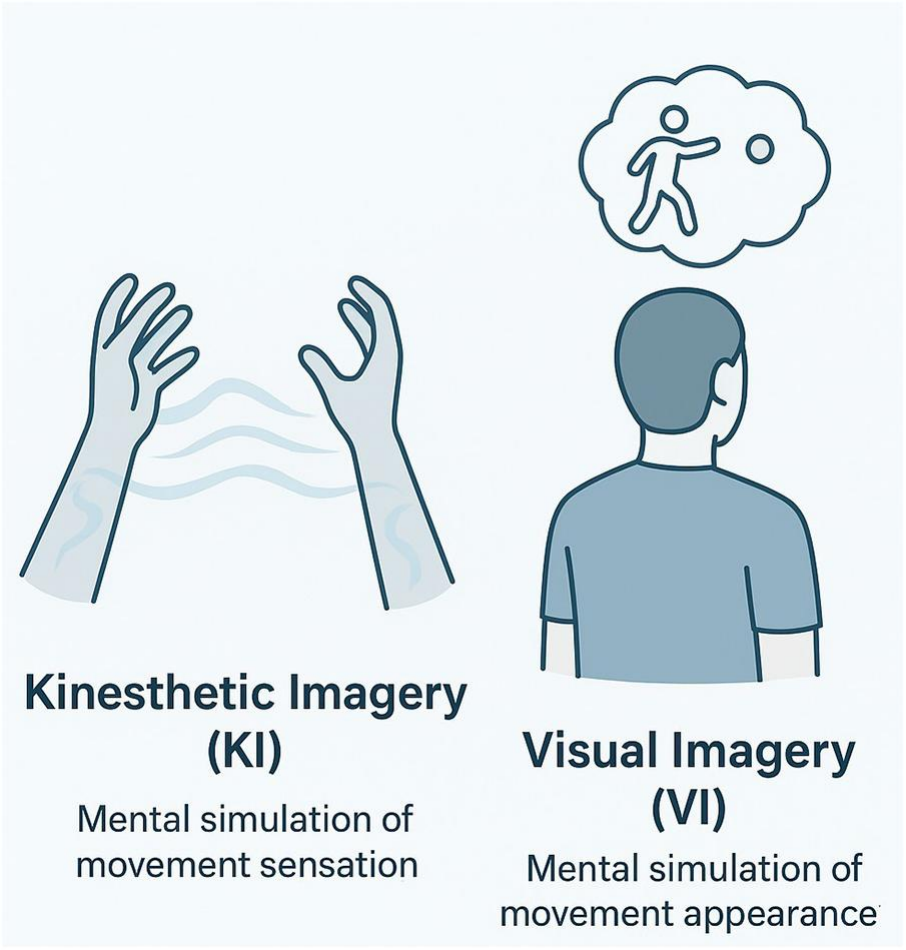
Types of motor imagery (MI). Kinesthetic imagery (KI) involves the first-person mental simulation of the sensation of movement. Visual imagery (VI) involves the third-person mental visualization of the appearance of movement.

Motor imagery can be broadly categorized into kinesthetic imagery (KI) and visual imagery (VI) (19). KI involves a first-person experience of movement-related sensations, such as force, tension, and changes in body position, and is closely associated with sensorimotor processing. In contrast, VI reflects a third-person perspective, emphasizing visual and spatial aspects of movement. These two forms of imagery may serve complementary roles in motor learning, with KI more closely linked to sensorimotor coupling and VI supporting spatial representation and movement planning (see Figure 2).

### 4.2 Neural mechanisms of motor imagery

Neuroimaging studies suggest that MI engages neural networks that overlap with those involved in motor execution, including the premotor cortex, supplementary motor area, parietal regions, and cerebellum (55). This overlap has been interpreted as supporting the “functional equivalence hypothesis,” which proposes e that imagined and executed movements share partially overlapping neural representations (56). Evidence from EEG fNIRS studies further indicates that MI is associated with activity changes in motor-related regions, reflecting processes related to motor preparation and coordination (57–60). Together, these findings provide a neurophysiological basis for considering MI as a potential tool for motor learning.

### 4.3 Theoretical models and mechanistic frameworks

Theoretically, the role of MI can be understood as involving processes of internal model activation and reinforcement (44). It can be conceptualized as a form of “motor simulation at a cognitive level”, which may engage predictive and corrective processes in the absence of overt movement. Among the numerous theories attempting to explain the nature of MI, two representative frameworks currently dominate: Motor Simulation Theory (MST) (61–63) and Motor Emulation Theory (MET) (64, 65).

Motor Simulation Theory (MST) proposes that motor imagery and motor execution rely on overlapping neural representations, such that imagined actions can be understood as internally simulated executions without overt movement (61–63). In contrast, MET adopts a computational neuroscience perspective, emphasizing the predictive function of the motor system. According to this theory, internal forward models generate predicted sensory consequences of actions, linking motor commands with expected (64, 65) perceptual outcomes.

**Figure 3:**
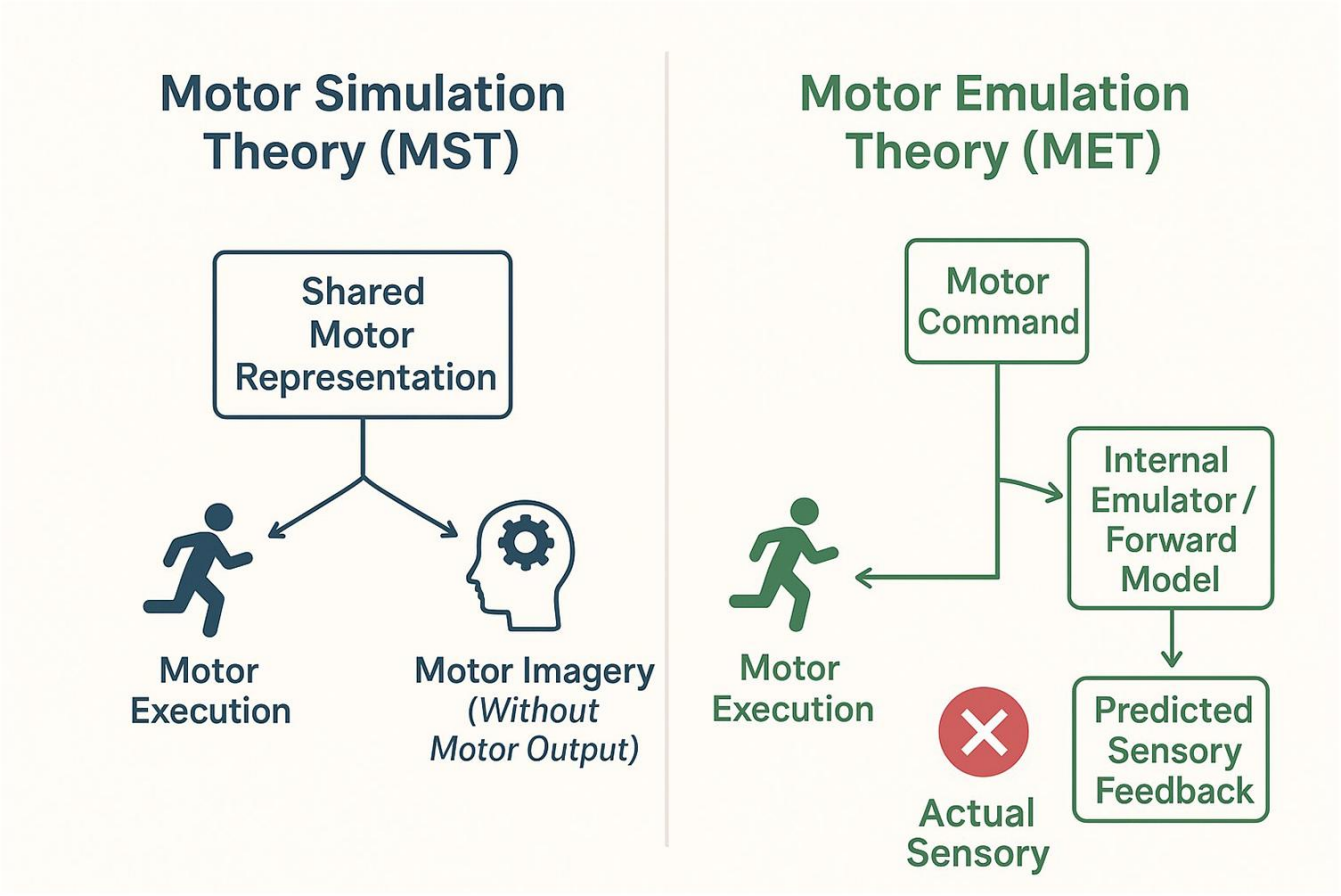
Theoretical frameworks explaining the mechanisms of motor imagery. (Left) Motor Simulation Theory (MST) posits that motor imagery and motor execution share identical underlying motor representations. (Right) Motor Emulation Theory (MET) emphasizes the role of an internal emulator, driven by a forward model, which predicts the sensory consequences of motor commands, creating a closed-loop simulation independent of movement execution.

In summary, MST emphasizes that motor system activity is driven by internal representations, focusing on “functional equivalence” at the mental simulation level. While MET posits that MI simultaneously simulates the dynamic interaction between motor and sensory systems, embodying dual simulation of movement and perception (see Figure 3) (62–65). Though differing in perspective, both theories reveal that motor imagery can be understood as an active, dynamic cognitive and neural process. Its core mechanism may involve coordination between movement planning and sensory prediction through internal mechanisms. Furthermore, both theories fundamentally converge on a key consensus: motor imagery is not a passive recall of actions but an active process of prediction and updating. Individuals continuously generate and refine sensory-motor pairings within imagery, potentially supporting perceptual-motor coupling and temporal aspects of movement coordination. For children with DCD, whose internal model generation and sensory feedback utilization are impaired, MI training serves as a cognition-driven rehabilitation approach. It facilitates the psychological reconstruction of movement temporal structures and representational accuracy, potentially supporting improvements in motor control.

## 5 Empirical evidence for motor imagery interventions in DCD

Among children with Developmental Coordination Disorder (DCD), motor imagery (MI) has been increasingly recognized as a potential intervention approach that may engage cognitive-motor processes and support neuroplasticity. Early studies primarily focused on examining whether DCD children possess intact motor imagery abilities, with many reporting impairments in imagery performance (8), while more recent research has explored whether structured MI-based interventions can still improve their motor function despite these imagery-related deficits. Key findings from recent intervention studies are summarized in Table 1.

**Table 1:** Summary of key empirical studies on motor imagery (MI) and combined action observation and motor imagery (AOMI) interventions in children with developmental coordination disorder (DCD).

Study (Author, Year)	Participants (DCD)	Intervention Design	Key Findings
Wilson et al. (66)	*N* = 36	Groups: MI vs. PMT vs. Waitlist	MI training was comparable to PMT in improving motor skill proficiency.
		Duration: 5 weeks	
		Frequency: 1x/week, 60 min	
Adams et al. (67)	*N* = 8	Groups: MI vs. CO-OP (Feasibility)	Parents and children reported significant improvements.
		Duration: 9 weeks	
		Frequency: 1x/week, 45min	Some children showed >2 SD improvement on MABC-2.
Scott et al. (68)	*N* = 24	Groups: AOMI (home-based) vs. Control	AOMI group showed 89% improvement in ADL tasks vs. 44% in control.
		Duration: 4 weeks	
		Frequency: 4x/week, 40min	

This table synthesizes findings from pivotal intervention studies. PMT, Perceptual-Motor Training; CO-OP, Cognitive Orientation to daily Occupational Performance; MABC-2, Movement Assessment Battery for Children-2; ADL, Activities of Daily Living.

In a representative randomized controlled trial, Wilson et al. (66) randomly assigned 36 children with DCD to a Motor Imagery group, a Perceptual–Motor Training (PMT) group, or a waiting list control group for a five-week intervention. The MI group underwent weekly 60-minute sessions combining action observation and mental imagery tasks. Results indicated that MI training produced improvements comparable to traditional PMT in motor skill proficiency; however, the relatively small sample size and short intervention duration limit the generalizability of these findings. Subsequently, Adams et al. (67) conducted a feasibility study grouping 8 children with DCD to receive either the MI intervention or the cognitive-oriented (CO-OP) intervention. Results suggested both children and parents in the MI group reported improvements in motor skills, with some children showing notable gains; however, the very small sample size and feasibility design limit the strength of the conclusions. Together, these studies suggest that MI interventions may achieve improvements comparable to traditional rehabilitation approaches; however, the evidence remains preliminary and should be interpreted with caution.

More recent evidence comes from a randomized controlled trial by Scott et al. (68). They employed a family-oriented, parent-led “Action Observation + Motor Imagery (AOMI)” training paradigm to intervene with children aged 7–12 years with DCD. After several weeks of training, the AOMI group showed greater improvements than the control group in daily living tasks such as tying shoelaces, using utensils, and stacking cups, with an improvement rate of 89% (control group: 44%). These results suggest that MI combined with action observation may facilitate transfer to daily living tasks; however, replication in larger and more diverse samples is needed.

A synthesis of existing evidence suggests that MI interventions are associated with generally positive short-term outcomes for children with DCD and may show transfer to real-life contexts. Its core advantages include: (1) engaging motor representation processes without requiring intensive physical exertion; (2) potentially enhancing effectiveness when combined with action observation or physical practice; and (3) showing good acceptability in preliminary studies and potential for home-based implementation. Despite generally positive findings, the current evidence base remains limited. Common issues include small sample sizes, variability in intervention protocols, differences in outcome measures, and a lack of longitudinal follow-up, which constrain the strength and comparability of findings.

## 6 Moderators and mechanisms of MI training efficacy

Although existing research suggests that motor imagery interventions may improve motor performance in children with developmental coordination disorder (DCD), training effects still vary considerably across individuals. This variability reflects the crucial moderating roles of cognitive, task-related, and psychological factors in the intervention.

At the cognitive level, children's understanding of imagery and their cognitive control abilities form the foundation for MI success. To effectively apply MI techniques in pediatric populations, it is important to ensure that individuals understand the concept of “imagining movements” and possess basic cognitive control. Spruijt et al. (69) noted in their review that motor imagery abilities in typically developing children develop steadily between ages 5 and 10, with imagery task difficulty closely correlating with movement complexity. Therefore, given that children with DCD commonly exhibit deficits in motor planning and executive function, a simple imagery assessment should determine the individual's training baseline before implementing MI interventions. Structured visual cues and verbal guidance should be provided during intervention to construct cognitive scaffolding.

At the training parameter level, intensity and frequency are key external variables influencing MI effectiveness. Existing research generally agrees that high-frequency, structured training may yield greater effects than single-session training. Wilson et al. (66) demonstrated that a 5-week intervention involving one 60-minute session per week significantly improved motor coordination. However, for children with DCD who have limited attentional resources, shorter, more frequent training sessions may be more effective. This perspective aligns with international clinical recommendations (9): intervention design must address both motor and non-motor functions in DCD children, including executive functions such as selective and sustained attention and inhibitory control. Moderate gamification and multisensory feedback can help sustain attention and reduce cognitive fatigue, thereby enhancing engagement.

Psychologically, motivation and self-efficacy are crucial for sustaining training gains. Children with DCD often exhibit low confidence when facing complex motor tasks, and MI offers a low-risk “mental rehearsal” pathway enabling them to build successful experiences in a safe environment. Research indicates that involving children in goal setting and self-assessment of progress significantly improves training adherence (67). Furthermore, self-modeling strategies, where children watch video recordings of themselves completing tasks, can further strengthen self-efficacy and proactive learning tendencies (70). Given that self-efficacy is often reduced in children with DCD, examining changes in self-efficacy and related psychosocial outcomes may represent an important dimension for evaluating the effectiveness of MI interventions in future research.

Attention and comorbid factors present key challenges in MI application. Attention-deficit/hyperactivity disorder (ADHD) frequently co-occurs with DCD, and its fluctuating attention impacts image generation and maintenance processes demanding high attentional control. Given the high rate of comorbid conditions in individuals with DCD, future studies should more systematically account for comorbidity in participant selection, subgroup analysis, and interpretation of intervention outcomes. Adams et al. (67) recommend including only individuals confirmed by occupational therapists to maintain attention. For such children, visual cues (e.g., video prompts) and eye-tracking feedback technology can effectively help them focus on critical movement phases (71), thereby sustaining higher consistency and engagement during MI training.

Finally, task selection and ecological relevance influence MI transfer effects. Training based on familiar or contextually meaningful daily activities (e.g., tying shoelaces, pencil grip, utensil use) fosters greater active engagement and cross-situation application. Scott et al.'s (68) home-based intervention study demonstrated that MI programs using real-life tasks exhibit higher ecological validity and transferability.

In summary, the efficacy of motor imagery interventions is unlikely to be determined by a single mechanism but results from the synergistic interaction of multiple individual and environmental factors. Future research should systematically compare the effects of different training frequencies, task types, and support strategies on treatment outcomes using larger samples and longitudinal designs. Additionally, it should explore how to integrate standardization and individualization of MI in school or home settings.

## 7 Pathways for technological advancement and intelligence in MI

With the rapid development of artificial intelligence and immersive interaction technologies, motor imagery interventions are increasingly being explored beyond traditional laboratory training toward more technology-supported and potentially scalable applications. Recent reviews focusing on DCD populations suggest that virtual reality (VR) and augmented reality (AR) have emerged as promising tools in neurorehabilitation, particularly for supporting cognitive processes such as mental imagery manipulation and motor planning. Despite growing interest in this area, only a limited number of studies have directly examined the effects of VR/AR-based interventions on mental imagery processes in children with DCD, indicating that the field remains at an early stage (72). However, related advances in motor rehabilitation, action observation, and movement science provide an important foundation for understanding how these technologies may support MI-related processes.

First, Virtual Reality (VR) technology may provide more immersive and controllable contextual support for MI training. By presenting movement tasks in a three-dimensional virtual environment, children can observe and imagine the movement process from a first-person perspective, potentially supporting sensorimotor integration (73). Notably, recent evidence from DCD populations provides more direct support for the role of VR in enhancing internal modeling and MI-related processes. A quasi-experimental study demonstrated that VR-based training significantly improved predictive internal modeling, as indexed by continuous relative phase (CRP), and facilitated the transfer of these improvements to object control skills, with effects retained at follow-up (74). Similarly, randomized controlled evidence indicates that VR interventions can improve motor imagery ability and predictive motor control in children with DCD (12). However, most supporting evidence still derives from adult or non-DCD populations, limiting direct generalization (75). VR systems can also provide visual cues that help integrate imagery with action execution, which may be particularly beneficial for children with attentional or motivational difficulties.

In addition to experimental studies, technology-assisted systems grounded in action observation and mirror neuron principles have been applied in rehabilitation contexts. These systems integrate action observation, motor imagery, and imitation-based execution within structured training protocols, and have been shown to improve fine motor skills and daily functioning in children with DCD (76). This suggests that MI-related mechanisms can be embedded within broader technology-supported intervention systems rather than implemented as isolated training components.

Second, wearable sensing devices and multimodal monitoring technologies may support the quantification and personalization of MI. Smart wristbands, motion capture systems, and electromyography (EMG) sensors can record physiological and behavioral data during MI training (77), enabling analysis of children's attentional focus, muscle tension changes, and movement rhythms. These objective metrics may serve as useful supplements to training evaluations and provide the data foundation for algorithm-based dynamic feedback (78). For instance, studies in adult and healthy pediatric populations have demonstrated that multimodal models integrating electroencephalography (EEG), functional near-infrared spectroscopy (fNIRS), or EMG signals have been shown to enhance MI decoding accuracy and task adaptability in adult or non-DCD populations (79, 80). However, their applicability to children with DCD remains largely unexplored, particularly in real-time adaptive intervention contexts.

In addition, AI-based approaches may enable more adaptive and personalized intervention strategies by dynamically adjusting task difficulty, feedback timing, and cueing strategies according to individual performance. A recent randomized controlled trial demonstrated that an AI-supported occupational therapy program significantly improved handwriting performance across multiple domains in children at risk for DCD (81). These improvements were attributed to real-time feedback and individualized training pathways, highlighting the potential of AI to optimize motor learning environments. However, current applications remain limited, and further research is needed to examine how AI can be effectively integrated with MI-based interventions.

Finally, mobile and cloud platform technologies may enhance the scalability and accessibility of MI interventions. Tablet- or smartphone-based MI training applications (e.g., gamified imagery training programs) enable long-term implementation at home, facilitating remote rehabilitation management through cloud-based monitoring and data analysis (82). Scott et al.'s (68) home-based intervention study has preliminarily validated the feasibility of such “home-directed MI protocols”. In the future, integrating these mobile systems with modules like VR and AI may potentially contribute to the development of more standardized and accessible rehabilitation ecosystems, providing integrated intervention support for children with DCD.

In summary, the convergence of MI with emerging technologies may represent a potential transition from traditional cognitive training to more technology-supported rehabilitation approaches. VR/AR may provide immersive scenarios and perceptual guidance, while wearable and AI systems may enable personalized adjustments and remote monitoring. However, current evidence remains limited and is largely derived from adult or non-DCD populations. Future research should therefore focus on systematically evaluating how these technologies can support MI-related cognitive mechanisms—such as imagery generation, predictive control, and sensorimotor integration—in children with DCD.

## 8 Current challenges and practical limitations

Although MI interventions have shown promising findings in theoretical experimental settings, the practical implementation of MI interventions among children with DCD faces multiple challenges. These primarily include methodological limitations, issues of individual variation, implementation barriers, and insufficient ecological validity.

### 8.1 Methodological limitations

Current research on MI interventions for children with DCD generally features small sample sizes and includes relatively few high-quality randomized controlled trials (RCTs) and long-term follow-up designs. Significant variations exist across studies in MI task types, assessment tools, and outcome measures, hindering comparability, synthesis and replicability of findings (21, 66, 67). Furthermore, the reliability and validity of imagery assessment tools (e.g., MIQ-C, VMIQ) in pediatric populations remain partially established (83), further limiting consistency in data interpretation. In addition, recent work has proposed structured experimental protocols to assess motor learning–related processes, such as action observation and imitation abilities (84), which may serve as more sensitive and mechanism-oriented outcome measures for evaluating changes following MI interventions.

### 8.2 Individual variability and cognitive load issues

Children with DCD exhibit widespread deficits in sustained attention, working memory, and executive function (5), making them more prone to fatigue, distraction, or strategic biases during MI training. Without adequate visual or semantic support, children may struggle to generate stable motor representations consistently, which may reduce the effectiveness of training. Particularly for younger children, the cognitive demands of MI may exceed their developmental capacity (69), highlighting the need for age- and cognition-specific, tiered training protocols.

### 8.3 Implementation and ecological validity

MI interventions often demand high levels of motivation and self-regulation, yet children with DCD frequently exhibit low self-efficacy and frustration due to repeated motor failures (14). Traditional static imagery training lacks real-time feedback, leading to diminished interest and poor adherence. Most MI interventions remain confined to laboratory settings, lacking integration with daily activities and functionally meaningful skills in children's daily lives (e.g., tying shoelaces, using chopsticks), which may limit transfer to real-world contexts.

Overall, MI interventions for DCD children have established a relatively strong conceptual and mechanistic foundation but still face important challenges in three areas: standardization, ecological validity, and individualization. Future research should establish a unified intervention evaluation framework, and explore integrated models combining multi-level data (neural, behavioral, environmental) to advance MI from “small-sample experiments” to “scalable practice”.

## 9 Future directions and integrative perspectives

Future DCD interventions may benefit from moving beyond single cognitive training modalities, toward a more integrated framework characterized by “multilevel integration, technology-driven approaches, and ecological scalability.”.

First, future research should leverage multilevel modeling to integrate cognitive representations, neuroplasticity, and behavioral performance within a unified analytical framework. For instance, combining EEG/functional imaging with MI training studies may help to clarify potential pathways linking cortical activation changes to motor improvements, thereby contributing to a more systematic understanding of mechanisms.

Second, MI may be integrated with multimodal information such as language, visual attention, sensorimotor coordination, participants' physical condition, age, and motivation to form integrated intervention protocols. These multimodal inputs may help characterize individual learning trajectories. Such models not only may enhance intervention precision but also serve as cognitive assessment tools to identify children with delayed responses or learning plateaus.

Finally, future efforts should encourage embedding MI interventions within natural settings, such as school physical education, family activities, and gamified platforms, while leveraging mobile monitoring and longitudinal tracking for sustained outcome evaluation. Thus, this approach may support a cycle of intervention, assessment, and feedback, propelling MI from research-based intervention toward ecological rehabilitation.

This integrated perspective underscores that MI may extend beyond the training of mental representations and may contribute to the development of more integrative rehabilitation approaches shaped by the convergence of cognitive science, neuroscience, and educational psychology.

## 10 Conclusion: MI as a key mechanism for early prediction and proactive intervention

This paper provides a narrative synthesis of the cognitive and representational deficits in children with Developmental Coordination Disorder (DCD), the mechanisms of Motor Imagery (MI) in intervention, empirical evidence, and influencing factors. It further explores the potential and practical challenges of integrating MI with emerging technologies. Overall, as a cognitive training method grounded in internal representation, MI shows promising effects not only in improving motor execution and planning abilities but also offers new perspectives for understanding the cognitive essence of DCD and its developmental intervention mechanisms.

Future directions involve expanding MI from a “single training modality” to an integrated “prediction-intervention” cognitive mechanism tool. Existing research indicates that children with DCD exhibit systematic biases in temporal matching, spatial consistency, and sensory feedback integration (17, 41), characteristics that may be detectable before clinical manifestation. Thus, quantifiable MI metrics may provide a potential basis for early risk identification and stratified intervention. Brief MI tasks or neurophysiological measurements may support early identification of potential motor coordination deficits and provide cognitive targets for proactive intervention.

Furthermore, by combining multimodal data across cognitive, neural, and behavioral levels, a closed-loop cognitive rehabilitation model spanning “early screening-intervention-tracking” may emerge, potentially contributing to a shift from passive compensation to active restructuring.

In summary, MI may serve not only as a means to improve motor function in children with DCD but also as a useful window for understanding and intervening in their cognitive impairments. Future efforts should focus on advancing mechanism research, standardizing methodologies, and developing intelligent implementation pathways to facilitate further integration of MI across cognitive science, rehabilitation engineering, and educational practice.
